# Alarm Fatigue and Nursing Performance Among Hospital Nurses in South Korea: A Cross-Sectional Study of Indirect Association Through Perceived Stress and Moderating Role of Patient Safety Culture

**DOI:** 10.3390/healthcare14121650

**Published:** 2026-06-10

**Authors:** Jungmi Kwon, Yoonjoo Kim

**Affiliations:** College of Nursing, Dongguk University WISE, Gyeongju 38066, Republic of Korea; onlyu2102@dongguk.ac.kr

**Keywords:** alarm fatigue, nurses, perceived stress, patient safety culture, nursing performance

## Abstract

**Highlights:**

**What are the main findings?**

- Alarm fatigue was associated with lower performance and higher perceived stress among hospital nurses.
- Perceived stress was indirectly associated with alarm fatigue and nursing performance, and patient safety culture buffered the stress–performance link.

**What are the implications of the main findings?**

- Alarm-related problems should be addressed through technical alarm management and support for nurses’ stress responses.
- Strengthening patient safety culture may help protect nursing performance under high job demands, although its buffering effect was modest.

**Abstract:**

**Background/Objectives**: Alarm fatigue is common in clinical settings and can impair nursing performance. Guided by the job demands–resources framework, this cross-sectional study examined the associations among alarm fatigue, nursing performance, perceived stress, and patient safety culture, including an indirect association through perceived stress and moderation by patient safety culture. **Methods**: Self-report questionnaires completed by 218 registered nurses from two general hospitals in South Korea were used to assess alarm fatigue, perceived stress, patient safety culture, and nursing performance. Data were analyzed using descriptive statistics, Pearson’s correlations, and Hayes’ PROCESS macro with 5000 bootstrap samples controlling for covariates. **Results**: Alarm fatigue was positively correlated with perceived stress and negatively correlated with nursing performance. Patient safety culture was positively correlated with nursing performance. Alarm fatigue was positively associated with perceived stress, and perceived stress was negatively associated with nursing performance. The indirect association through perceived stress was significant. Patient safety culture moderated the association between perceived stress and nursing performance. The index of the moderated indirect association was significant. **Conclusions**: These findings suggest that alarm-related problems should be addressed in clinical and assistive nursing practice by integrating strategies aimed at reducing unnecessary alarms, strengthening training in alarm prioritization and interruption management, supporting nurses’ stress responses, and promoting a patient safety culture characterized by open communication and teamwork. Considering the cross-sectional design of this study, the findings should be interpreted as associations, rather than causal evidence.

## 1. Introduction

Alarms generated by medical devices, such as physiological monitors, mechanical ventilators, and infusion pumps, are essential safety mechanisms that support the early detection of patient deterioration and timely clinical intervention in contemporary hospital settings [1,2]. However, such environments are often characterized by a high frequency of nonactionable, technically triggered, or low-priority alarms that do not necessarily require an immediate response [3,4,5]. Repeated exposure to such alarms may create persistent attentional demands, contribute to sensory desensitization and delayed responses, and fragment the workflow of frontline clinicians [3,4,5,6,7]. This phenomenon, commonly referred to as alarm fatigue, may reduce responsiveness to clinically relevant alarms and interfere with safe and efficient care delivery, making it a critical patient safety concern [6,7]. Nurses are particularly vulnerable to this problem because they are the healthcare professionals most frequently exposed to alarms and are most often required to interpret alarm relevance and respond in real time in alarm-intensive settings, such as general wards, intensive care units (ICUs), and emergency departments [8,9].

Nursing performance, which refers to nurses’ integration of professional knowledge, technical skills, clinical judgment, communication, and coordination to deliver safe and effective patient care, may be directly affected by alarm fatigue [7,10]. From a clinical and assistive practice perspective, repeated alarms are not merely background noise. They require nurses to interrupt ongoing care, reassess priorities, distinguish between meaningful and nonactionable signals, and maintain patient safety while managing competing tasks. These interruptions may increase cognitive load, compromise concentration and situational awareness, and disrupt care coordination [11,12,13]. Although prior studies have mainly addressed alarm frequency, response behavior, technical optimization, and safety incidents, less attention has been paid to nursing performance as a multidimensional clinical outcome [14,15].

The stress-related association between alarm fatigue and nursing performance also requires consideration [10,12,14,16]. In alarm-intensive practice, nurses may consider repeated exposure to alarms to be excessive, unpredictable, or difficult to control, particularly alongside competing clinical demands [11,13,16]. Such appraisals may contribute to perceived stress, which can impair concentration, decision-making, emotional regulation, work engagement, and overall work functioning in demanding clinical environments [10,12,14,16]. Thus, perceived stress may indirectly link alarm fatigue to decreased nursing performance.

Burnout is also clinically relevant to this context. Burnout, a work-related syndrome associated with chronic workplace stress that has not been successfully managed, is particularly important in nursing settings characterized by high workloads, limited control, emotional demands, sustained vigilance, and insufficient organizational or social resources [17]. Evidence from critical care nursing, prison and correctional nursing, and broader hospital nursing contexts indicates that burnout is associated with coping resources, job satisfaction, social support, alarm-related burden, and work functioning [7,9,18,19,20,21,22]. Although burnout was not measured in this study, the literature situates alarm fatigue within the broader clinical problem of stress-intensive nursing practice.

Organizational context also shapes nursing performance. Patient safety culture refers to the shared values, norms, communication patterns, teamwork, leadership, and learning practices that prioritize patient safety in healthcare organizations [16,23]. A positive patient safety culture may support nursing performance by promoting open communication, mutual support, managerial responsiveness, nonpunitive reporting, and learning from errors [16,23,24]. In this study, nurses’ perceived patient safety culture was conceptualized as individual-level perceptions of an organizational resource that may support nursing performance and attenuate the negative association between perceived stress and nursing performance.

Several gaps remain in the literature. Alarm fatigue, perceived stress, patient safety culture, and nursing performance have often been examined separately rather than within a single explanatory model [8,13,14]. Notably, patient safety culture has rarely been incorporated as a potential protective organizational resource in the alarm fatigue–stress–performance association [15,24]. Moreover, alarm-related research has often focused on highly monitored settings, such as ICUs, although alarm frequency [8,25,26,27], workflow interruptions, and job demands vary across hospital units. Therefore, an integrated model is required across diverse nursing settings.

The job demands–resources (JD-R) model explains how job demands and job resources jointly shape strain and work functioning [28,29]. Job demands require sustained cognitive or emotional effort and may increase strain, whereas job resources support performance and buffer adverse demand-related effects. In alarm-intensive nursing practice, repeated alarm exposure represents a job demand, and perceived stress reflects a strain response when these demands are appraised as excessive or difficult to control. Nurses’ perceived patient safety culture may function as an organizational resource that supports performance under stressful conditions. Thus, the JD-R theory supports the integrated model of this study.

The primary objective of this study was to examine the association between alarm fatigue and nursing performance among hospital nurses in South Korea. The secondary objectives were to examine whether this is an indirect association through perceived stress, nurses’ perceived patient safety culture moderates the association between perceived stress and nursing performance, and the indirect association differs according to the level of perceived patient safety culture. The hypotheses were as follows:

**H1.**  *Alarm fatigue is negatively associated with nursing performance*.

**H2.**  *Alarm fatigue is positively associated with perceived stress, and perceived stress is negatively associated with nursing performance. The association between alarm fatigue and nursing performance is statistically consistent with an indirect association through perceived stress*.

**H3.**  *Nurses’ perceived patient safety culture moderates the association between perceived stress and nursing performance, such that the negative association is weaker when perceived patient safety culture is higher*.

**H4.**  *The indirect association between alarm fatigue and nursing performance through perceived stress differs according to the level of nurses’ perceived patient safety culture*.

## 2. Materials and Methods

### 2.1. Study Design

This cross-sectional, correlational study examined the association between alarm fatigue and nursing performance among hospital nurses and evaluated whether this association was statistically consistent with an indirect association through perceived stress, and whether patient safety culture moderated the association between job stress and nursing performance. This study was guided by the JD-R theory [28,29], in which alarm fatigue was specified as the job demand, perceived stress as the strain response, patient safety culture as the organizational resource, and nursing performance as the primary outcome. This study was reported following the STROBE checklist for cross-sectional studies, which is provided as Supplementary Table S1.

### 2.2. Participants and Data Collection

Convenience sampling was used to collect data between August and September 2025 from registered nurses working at two general hospitals in G City, Republic of Korea. Eligible participants were nurses with at least six months of clinical experience [30]. Outpatient nurses with limited exposure to physiological monitoring alarms and nurses who were on leave during the data collection period (e.g., parental leave or sick leave) were excluded.

A total of 240 questionnaires were distributed and 229 were returned (response rate: 95.4%). Of these, 11 questionnaires were excluded because they did not meet the eligibility criteria or one or more variable scores could not be computed owing to insufficient item responses. Accordingly, data from 218 participants were included in the final analysis. Participation was voluntary, and written informed consent was obtained from all participants after they had received an explanation of the study purpose and procedures.

The sample size was determined using G*Power 3.1.9.7 [31] for the multiple regression model. The number of predictor terms was specified to account for the main variables, the interaction term, and dummy-coded covariate categories and obtain a conservative estimate for the fully adjusted model. Assuming an alpha level of 0.05, an effect size of f^2^ = 0.15, a power of 0.80, and 20 predictor terms, the minimum required sample size was estimated to be 157. Considering a potential attrition rate of 20%, the target sample size was 197. Thus, the final sample size of 218 provided adequate statistical power.

This study was approved by the Institutional Review Board of the authors’ affiliated university (approval no. DGU 20250022) and was conducted in accordance with the Declaration of Helsinki. All data were anonymized, and participants’ personal information was used only for research purposes. The study data were stored in encrypted files on a password-protected computer accessible only to the principal investigator and co-investigators. Data will be retained for three years after study completion, after which electronic files will be permanently deleted and printed materials will be shredded.

### 2.3. Measures

#### 2.3.1. Alarm Fatigue

Alarm fatigue was measured using the Charité Alarm Fatigue Questionnaire developed by Wunderlich et al. [6]. This study used the Korean version translated and validated by Lee and Choi [32]. This scale comprises nine items: five for assessing alarm-related stress and four for assessing alarm-related coping. Because the alarm coping items reflect the positive aspects of alarm management, they were reverse-coded before calculating the total score. Each item is rated on a 5-point Likert scale (1 = strongly disagree, 5 = strongly agree), and the total score ranges from 9 to 45, with higher scores indicating a higher level of alarm fatigue. In the present sample, Cronbach’s α was 0.91.

#### 2.3.2. Perceived Stress

Perceived stress was operationally defined as nurses’ subjective stress appraisal under alarm-related job-demand conditions. Perceived stress was measured using the 10-item Perceived Stress Scale (PSS-10), originally developed by Cohen [33]. This study used the Korean version of the PSS, which was validated by Park and Seo (2010) [34]. The PSS-10 assesses the degree to which individuals perceived situations during the past month as unpredictable, uncontrollable, and overly burdensome. Each item was administered using a 1–5 response format to ensure consistency with the other study instruments. Item responses were linearly recoded to the original 0–4 metric before calculating the total score to maintain comparability with the standard PSS scoring system. The four positively worded items were reverse-coded before scoring. Total scores ranged from 0 to 40, with higher scores indicating higher perceived stress. In the present sample, Cronbach’s α was 0.93.

#### 2.3.3. Nursing Performance

Nursing performance was measured using the Six-Dimension Scale of Nursing Performance developed by Schwirian [35]. This scale comprises 52 items across six subdomains: leadership (5 items), critical care (7 items), teaching/collaboration (11 items), planning/evaluation (7 items), interpersonal relations/communication (12 items), and professional development/completion of nursing care (10 items). A frequency-based 5-point Likert scale (1 = never, 5 = always) was used to assess the frequency of each behavior performed by the nurses. Total scores ranged from 52 to 260, with higher scores indicating better nursing performance. This study used the Korean version translated and validated through translation and back-translation procedures by Park et al. [36]. In the present sample, Cronbach’s α was 0.99.

#### 2.3.4. Patient Safety Culture

Patient safety culture was measured using the Korean version of the original Hospital Survey on Patient Safety Culture (HSOPSC Version 1.0), originally developed by the Agency for Healthcare Research and Quality (AHRQ) and adapted to Korean by Kim et al. [37,38]. Although the AHRQ subsequently released the SOPS Hospital Survey Version 2.0, this study used a previously validated Korean version based on HSOPSC Version 1.0 because it was considered appropriate for the study context and analytical purposes. Among the 44 items in the Korean version, one item assessing patient safety event reporting experiences during the previous year was treated as a separate descriptive item and not included in the patient safety culture score. Thus, 43 items were included in the main analysis. Items were rated on a 5-point Likert scale, and negatively worded items were reverse-coded, so that higher scores indicated more positive perceptions of patient safety culture. Patient safety culture was modeled as an individual-level continuous moderator rather than as a unit- or hospital-level benchmarking indicator. Thus, the summed score of the 43 scored items was used for the regression analyses. In the present sample, Cronbach’s α was 0.983.

### 2.4. Statistical Analysis

Data were analyzed using IBM SPSS Statistics version 29 (IBM Corp., Armonk, NY, USA) and Hayes’ PROCESS macro version 4.2 [39]. Participants’ general characteristics and study variables were summarized using descriptive statistics. Bivariate associations among the main variables were examined using Pearson’s correlation coefficients.

PROCESS Model 4 was applied to examine whether alarm fatigue (X) and nursing performance (Y) were indirectly associated through perceived stress (M). Subsequently, PROCESS Model 14 was used to test whether patient safety culture (W) moderated the association between perceived stress (M) and nursing performance (Y) and whether the indirect association varied according to the level of patient safety culture. All continuous variables were mean-centered before creating the interaction term, and heteroscedasticity-consistent standard errors (HC3) were used.

Conditional effects were estimated at three levels of patient safety culture (−1 standard deviation (SD), mean, and +1 SD) to examine the direction and magnitude of the moderating effect. Covariates were selected a priori based on previous studies and theoretical relevance, and included education level, total clinical experience, position, current ward, and shiftwork status [40,41,42]. Categorical covariates were dummy-coded and entered into the models using the following reference categories: education level (4-year college graduate), position (staff nurse), current ward (general ward), and shiftwork status (yes).

A high correlation between alarm fatigue and perceived stress was anticipated. Therefore, multicollinearity was assessed in the regression models, including the main predictors, using the tolerance and variance inflation factor (VIF). No serious multicollinearity concerns were assumed when the tolerance was greater than 0.10, and the VIF was less than 5. The results are reported as unstandardized regression coefficients (B), standard errors (SEs), *p*-values, and 95% confidence intervals (CIs) [43]. The significance of the indirect and conditional indirect effects was tested using 5000 bootstrap samples, and the effects were considered statistically significant when the 95% bootstrap CI did not include zero. Two-tailed *p*-values < 0.05 were considered to indicate statistically significant direct effects. This study used cross-sectional data; therefore, the findings were interpreted as statistical associations, rather than causal effects.

**Figure 1:**
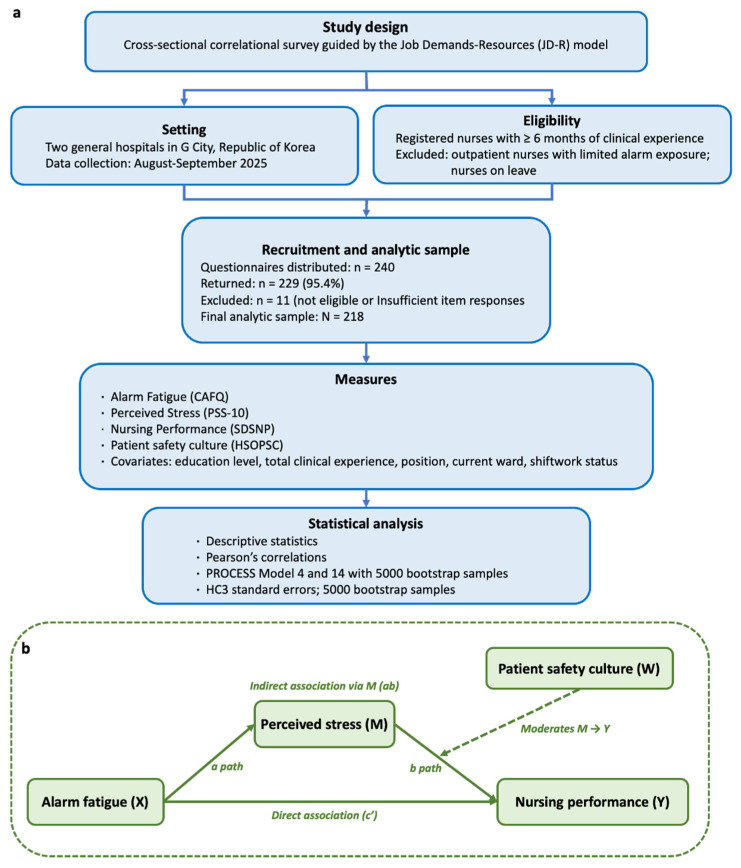
Summary flowchart of the study design, participant recruitment, measures, and analytic framework. Note. (**a**) Study flow presents the study design, setting, eligibility criteria, recruitment process, final analytic sample, measures, and statistical analysis. (**b**) Analytic framework presents the hypothesized indirect association between alarm fatigue and nursing performance through perceived stress and the moderating role of patient safety culture in the association between perceived stress and nursing performance. PSS-10 = 10-item Perceived Stress Scale; PROCESS = Hayes’ PROCESS macro. X = alarm fatigue; M = perceived stress; W = patient safety culture; Y = nursing performance.

Figure 1 summarizes the study design, participant recruitment process, measures, statistical analysis, and analytic framework.

## 3. Results

### 3.1. Participant Characteristics and Descriptive Statistics

The final analysis included 218 hospital nurses. The participants had a mean age of 34.97 years (SD = 8.06; range, 22–55 years), and most were women (95.4%). The majority had graduated from a 4-year college program (78.4%) and were staff nurses (86.2%). The mean total clinical experience was 132.72 months (SD = 98.88), and the mean current ward experience was 34.59 months (SD = 31.03). The most common work units were comprehensive nursing care units (33.9%), general wards (25.2%), and ICUs (23.4%). Most participants performed shiftwork (86.7%) and perceived their health status as fair or better (98.6%).

Regarding the study variables, the mean scores were 31.98 (SD = 8.18; range, 9–44) for alarm fatigue, 26.09 (SD = 9.11; range, 2–40) for perceived stress, 152.92 (SD = 37.50; range, 64–204) for patient safety culture, and 180.41 (SD = 48.53; range, 78–259) for nursing performance (Table 1).

### 3.2. Correlations Among Study Variables

Pearson correlation analyses showed that alarm fatigue was positively and significantly correlated with perceived stress (r = 0.876, *p* < 0.001) and negatively and significantly correlated with nursing performance (r = −0.665, *p* < 0.001). Perceived stress demonstrated a significant negative correlation with nursing performance (r = −0.697, *p* < 0.001). Patient safety culture was positively and significantly correlated with nursing performance (r = 0.506, *p* < 0.001) but not significantly correlated with alarm fatigue or perceived stress. Total clinical experience was negatively and significantly correlated with alarm fatigue (r = −0.356, *p* < 0.001) and perceived stress (r = −0.404, *p* < 0.001) and positively and significantly correlated with nursing performance (r = 0.524, *p* < 0.001) and patient safety culture (r = 0.432, *p* < 0.001) (Table 2).

Despite the high correlation between alarm fatigue and perceived stress, the two variables were retained as conceptually distinct. Alarm fatigue was assessed using an alarm-specific instrument that captures stress and coping difficulties related to repeated exposure to clinical alarms, whereas perceived stress was assessed using a general stress-appraisal instrument that measures the extent to which individuals perceived situations during the previous month as unpredictable, uncontrollable, and overly burdensome. Therefore, the high correlation was interpreted as a strong association between alarm-specific job demand and general strain response within the JD-R framework, rather than as evidence that the two constructs were identical. Multicollinearity diagnostics were also performed to assess model stability, and the results remained within acceptable limits (VIF = 4.374–4.402, tolerance ≥ 0.227).

### 3.3. Indirect Association Through Perceived Stress

PROCESS Model 4 was applied to examine whether the association between alarm fatigue and nursing performance was statistically consistent with an indirect association through perceived stress after controlling for education level, total clinical experience, position, current ward, and shiftwork status. Alarm fatigue was positively associated with perceived stress (B = 0.865, *p* < 0.001), and perceived stress was negatively associated with nursing performance (B = −1.819, *p* = 0.001). The total effect of alarm fatigue on nursing performance was significant (B = −2.846, *p* < 0.001), and the direct effect remained significant after perceived stress was included in the model (B = −1.271, *p* = 0.021).

**Figure 2:**
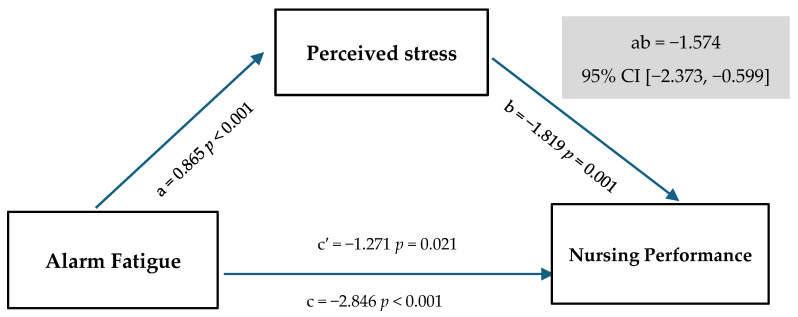
Indirect association through perceived stress in the relationship between alarm fatigue and nursing performance. Note: a = effect of alarm fatigue on perceived stress; b = effect of perceived stress on nursing performance; c = total effect of alarm fatigue on nursing performance; c′ = direct effect of alarm fatigue on nursing performance, controlling for perceived stress; ab = indirect effect; CI = confidence interval; p = significance level.

Bootstrap analysis based on 5000 samples indicated that the indirect effect of alarm fatigue on nursing performance through perceived stress was significant (ab = −1.574, 95% bootstrap CI [−2.373, −0.599]). Thus, the findings were consistent with a significant indirect association through perceived stress, while a direct association persisted. The magnitude of the indirect pathway accounted for approximately 55.3% of the total effect (Figure 2).

### 3.4. Moderating Effect of Patient Safety Culture and Moderated Indirect Association

PROCESS Model 14 was applied to examine whether patient safety culture moderated the association between perceived stress and nursing performance (Table 3). Alarm fatigue was positively associated with perceived stress (B = 0.865, SE = 0.040, t = 21.853, *p* < 0.001). In the nursing performance model, perceived stress was negatively associated with nursing performance (B = −3.060, SE = 0.447, t = −6.865, *p* < 0.001), whereas patient safety culture was positively associated with nursing performance (B = 0.430, SE = 0.075, t = 5.761, *p* < 0.001). The interaction between perceived stress and patient safety culture was significant (B = 0.023, SE = 0.008, t = 2.771, *p* = 0.006), indicating that the negative association between perceived stress and nursing performance was weaker at higher levels of patient safety culture.

In contrast to Model 4, the direct effect of alarm fatigue on nursing performance was not significant in Model 14 (B = −0.511, SE = 0.438, t = −1.165, *p* = 0.245). The model explained 73.6% of the variance in nursing performance (R^2^ = 0.736), while the interaction term contributed an additional 0.9% of the explained variance (ΔR^2^ = 0.009).

**Figure 3:**
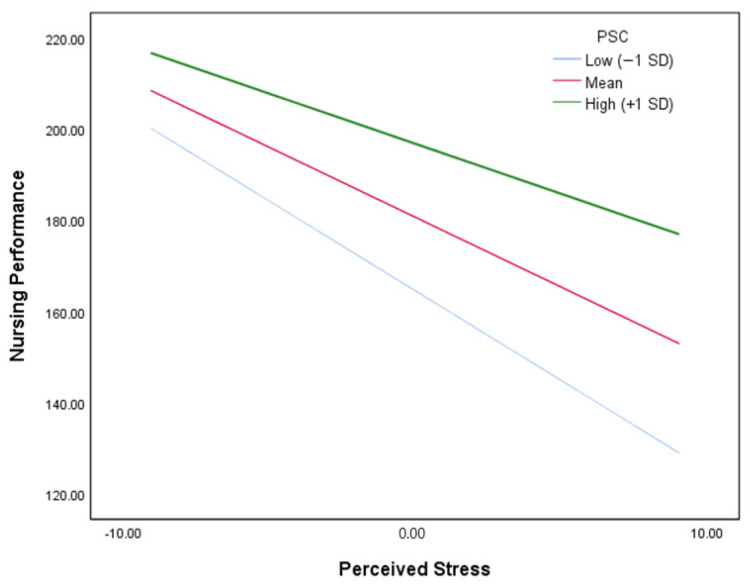
Conditional effects of perceived stress on nursing performance at different levels of patient safety culture.

Simple slope analyses showed that the negative association between perceived stress and nursing performance was strongest when patient safety culture was low (−1 SD: B = −3.935) and weakest when it was high (+1 SD: B = −2.197) (Table 4; Figure 3). The index of the moderated indirect association was significant (index = 0.023, BootSE = 0.007, 95% bootstrap CI [0.007, 0.037]), indicating that the magnitude of the indirect association between alarm fatigue and nursing performance through perceived stress varied according to the level of patient safety culture.

## 4. Discussion

### 4.1. Main Findings and Interpretation

This study examined alarm fatigue, perceived stress, patient safety culture, and nursing performance within an integrated framework in hospital nurses. Overall, the findings indicated that alarm fatigue was associated with lower nursing performance, which was statistically consistent with an indirect association through perceived stress, and nurses’ perceived patient safety culture attenuated the negative association between perceived stress and nursing performance. These findings support the usefulness of the JD-R framework [28,29] as an organizational perspective for understanding how alarm-related demands, strain responses, and organizational resources may be associated with nursing performance in alarm-intensive clinical settings. However, this was a cross-sectional study, and the findings should be interpreted as statistical associations rather than evidence of causal mechanisms.

A key implication of this study is that alarm fatigue should be understood not only as a technical or equipment-related issue but also as a clinically meaningful work-related burden. Repeated alarms interrupt ongoing tasks, require rapid prioritization, and demand sustained vigilance [3,4]. These features may help explain why alarm fatigue is associated with lower nursing performance [8,9,10,11,12,26]. This study’s findings extend those of previous alarm-related research by linking alarm fatigue to nursing performance as a multidimensional clinical outcome.

The findings also suggest that perceived stress may be part of the statistical association between alarm fatigue and nursing performance [18,44]. Alarm-related demands that are repeatedly experienced as excessive, unpredictable, or difficult to control may contribute to subjective stress appraisal, which can interfere with concentration, judgment, emotional regulation, and work engagement [26]. This interpretation is consistent with the health impairment process of the JD-R framework, in which sustained demands are associated with strain responses and poorer work functioning [28,29]. However, considering that perceived stress was measured using the PSS-10 [33], the construct should be understood as a general subjective stress appraisal rather than a narrowly defined occupational stress construct.

The high correlation between alarm fatigue and perceived stress suggests that nurses who experienced greater alarm fatigue also tended to report higher perceived stress. However, these constructs should not be interpreted as being interchangeable. Alarm fatigue reflects an alarm-specific occupational burden measured using an alarm-specific instrument, whereas perceived stress reflects a broader subjective appraisal of stress measured using a general stress appraisal instrument [6,45]. Nevertheless, the high correlation indicates substantial empirical proximity, and future studies should examine the discriminant validity of these constructs using approaches such as confirmatory factor analysis or heterotrait–monotrait ratio analysis [46,47,48].

Another notable finding is that patient safety culture moderated the association between perceived stress and nursing performance. Although patient safety culture was not significantly associated with alarm fatigue or perceived stress at the bivariate level, it attenuated the negative association between perceived stress and performance. This pattern suggests that patient safety culture may not necessarily reduce perceived stress. Instead, it may influence the strength of the association between perceived stress and impaired performance. This interpretation is consistent with the JD-R framework [28,29], which posits that job resources may buffer the negative impact of job demands and strain responses on work-related outcomes [49]. However, the buffering role of patient safety culture should be interpreted conservatively. Although the interaction term was significant, its incremental explanatory power was modest in this study. Therefore, patient safety culture should be viewed as one component of a broader support system rather than a standalone solution.

### 4.2. Perspectives for Clinical and Assistive Nursing Practice

From a clinical and assistive nursing practical perspective, these findings suggest that alarm fatigue should be addressed as an issue related not only to device management but also to clinical workflow and nursing performance [50,51]. Although reducing unnecessary alarms and optimizing alarm parameters remain important, these strategies may be insufficient if not accompanied by support for nurses’ cognitive and emotional workloads [50,51,52]. Educational programs should strengthen nurses’ ability to prioritize alarms, manage interruptions, distinguish clinically meaningful alarms from nonactionable signals, and cope with stress under high-demand conditions [51,52].

Organizational strategies are also required. Supportive leadership, open communication, nonpunitive reporting, teamwork, and feedback-based learning may help nurses maintain performance when perceived stress is high [53,54]. The moderating effect of patient safety culture was significant but modest, indicating that patient safety culture should be viewed as one component of a broader support system rather than as a standalone solution. Furthermore, nurses with less clinical experience may require particular attention because greater clinical experience was associated with lower alarm fatigue and perceived stress, as well as better nursing performance. Although this study did not measure burnout, the findings suggest that alarm-related burden and perceived stress should be considered in broader efforts to prevent stress accumulation and burnout risk in alarm-intensive clinical settings [20,21].

### 4.3. Limitations

This study had several limitations. First, the participants were recruited using convenience sampling from two general hospitals in one city in South Korea, which limits the generalizability of the findings. Therefore, the results may not fully represent nurses working in other hospital types, regions, countries, or clinical specialties. Differences in alarm systems, staffing levels, unit culture, nurse-to-patient ratios, patient acuity, and institutional safety practices may influence the associations between alarm fatigue, perceived stress, patient safety culture, and nursing performance. Second, the cross-sectional design precludes conclusions regarding temporal ordering or causality. Therefore, the indirect and moderated indirect associations observed in this study should be interpreted as patterns of statistical association, rather than as evidence of causal mechanisms. Third, all variables were measured using self-report questionnaires at a single time point, which may have introduced common method and social desirability biases. Fourth, the study did not include objective indicators, such as alarm logs, alarm density, nurse-to-patient ratios, patient acuity, supervisor-rated performance, and patient safety outcomes. Fifth, burnout, sleep quality, and workload were not fully assessed, although these factors may be relevant in alarm-intensive nursing environments. Finally, the high correlation between alarm fatigue and perceived stress indicates that future studies should examine the discriminant validity of these constructs using approaches such as confirmatory factor analysis or heterotrait–monotrait ratio analysis.

## 5. Conclusions

Alarm fatigue was associated with lower nursing performance among hospital nurses, which was statistically consistent with an indirect association through perceived stress. Nurses’ perceived patient safety culture attenuated the negative association between perceived stress and nursing performance. However, the magnitude of this buffering effect was modest. Therefore, patient safety culture should be understood as one supportive organizational factor rather than a sufficient solution to the consequences of alarm-related burden. The use of a cross-sectional design precludes the interpretation of the findings as evidence of causal mediation or moderation effects. Nevertheless, the results suggest that alarm-related problems in nursing practice may require integrated approaches that combine alarm management, support for nurses’ stress responses, and organizational efforts to strengthen patient safety culture. Future longitudinal and multilevel studies are required to confirm the temporal ordering of these associations and examine whether improvements in alarm management and safety culture contribute to better nursing performance.

## Figures and Tables

**Table 1:** General participant characteristics and descriptive statistics of the study variables (N = 218).

Variable		n (%)	M ± SD	Min–Max
Gender	Man	10 (4.6)		
	Woman	208 (95.4)		
Age (year)			34.97 ± 8.06	22–55
Education level	3-year college graduate	37 (17.0)		
	4-year college graduate	171 (78.4)		
	Master’s degree or higher	10 (4.6)		
Total clinical experience (months)			132.72 ± 98.88	6–480
Current ward experience (months)			34.59 ± 31.03	2–198
Position	Staff nurse	188 (86.2)		
	Charge nurse	22 (10.1)		
	Manager	8 (3.7)		
Monthly income (Won)	2,500,000–3,499,999	67 (30.7)		
	3,500,000–4,499,999	71 (32.5)		
	≥4,500,000	80 (36.7)		
Marital status	Single	116 (53.2)		
	Married	102 (46.8)		
Having children	Yes	79 (36.2)		
	No	139 (63.8)		
Current ward	Comprehensive nursing care unit	74 (33.9)		
	General ward	55 (25.2)		
	Intensive care unit	51 (23.4)		
	Emergency room	23 (10.6)		
	Operation room	9 (4.1)		
	Hemodialysis unit	4 (1.8)		
	Other	2 (0.9)		
Shift work	Yes	189 (86.7)		
	No	29 (13.3)		
Perceived health	Good	87 (39.9)		
	Fair	128 (58.7)		
	Poor	3 (1.4)		
Alarm fatigue			31.98 ± 8.18	9–44
Perceived stress			26.09 ± 9.11	2–40
Patient safety culture			152.92 ± 37.50	64–204
Nursing performance			180.41 ± 48.53	78–259

Note: Other includes nurses working in the delivery room and nursery; n = number; % = percentage; M = mean; SD = standard deviation; min = minimum; max = maximum.

**Table 2:** Pearson correlations among the study variables (N = 218).

Variables	1	2	3	4	5
	r (*p*)	r (*p*)	r (*p*)	r (*p*)	r (*p*)
1. Alarm Fatigue	1				
2. Perceived Stress	0.876	1			
	(<0.001)				
3. Nursing Performance	−0.665	−0.697	1		
	(<0.001)	(<0.001)			
4. Patient Safety Culture	−0.087	−0.041	0.506	1	
	(0.199)	(0.550)	(<0.001)		
5. Total Clinical Experience	−0.356	−0.404	0.524	0.432	1
	(<0.001)	(<0.001)	(<0.001)	(<0.001)	

Note: r = Pearson’s correlation coefficient; *p* = significance level. Values in parentheses indicate *p*-values.

**Table 3:** Moderated indirect association analysis (N = 218).

	Perceived Stress (M)				Nursing Performance (Y)			
	B	SE	t	*p*	B	SE	t	*p*
Alarm Fatigue (X)	0.865	0.040	21.853	*p* < 0.001	−0.511	0.438	−1.165	0.245
Perceived Stress (M)					−3.060	0.447	−6.865	*p* < 0.001
Patient Safety Culture (W)					0.430	0.075	5.716	*p* < 0.001
M × W					F = 7.680, *p* = 0.006			
R^2^	0.803				0.736			

Note: X = independent variable; Y = outcome variable; M = mediator; W = moderator; B = unstandardized regression coefficient; SE = standard error; t = t-value; *p* = significance level; R^2^ = coefficient of determination. All continuous variables were mean-centered before creating the interaction term (M × W).

**Table 4:** Conditional effects of perceived stress on nursing performance at patient safety culture levels and the index of moderated indirect association (N = 218).

**Patient Safety Culture Level**	**B**	**SE**	**t**	***p***	**LLCI**	**ULCI**
−37.496 (−1 SD)	−3.935	0.597	−6.593	*p* < 0.001	−5.111	−2.758
0.000 (M)	−3.066	0.447	−6.865	*p* < 0.001	−3.946	−2.185
37.496 (+1 SD)	−2.197	0.489	−4.491	*p* < 0.001	−3.161	−1.232
**Conditional indirect effect**	**Index**	**BootSE**		**LLCI**		**ULCI**
X → M → Y	0.023	0.007		0.007		0.037

Note: X = independent variable; Y = outcome variable; M = mediator; SD = standard deviation; B = unstandardized regression coefficient; SE = standard error; BootSE = bootstrap standard error; t = t-value; *p* = significance level; LLCI = lower limit confidence interval; ULCI = upper limit confidence interval. The arrows indicate the hypothesized mediation pathway: X → M → Y denotes the indirect association of alarm fatigue with nursing performance through perceived stress. All continuous variables (perceived stress and patient safety culture) were mean-centered before the analysis.

## Data Availability

The data presented in this study are available on request from the corresponding author due to ethical and privacy restrictions related to human participant data.

## Supplementary Materials

The following supporting information can be downloaded at: https://www.mdpi.com/article/10.3390/healthcare14121650/s1, Supplementary Table S1: STROBE Statement—Checklist of items that should be included in reports of cross-sectional studies.
